# Introducing walkable cities as a Public Health intervention: Vlatka Matkovic

**DOI:** 10.1093/eurpub/ckac129.077

**Published:** 2022-10-25

**Authors:** V Matkovic, M Jevtic, MP Kusturica, C Bouland, M Jukovic, D Stojanovic

**Affiliations:** 1 Health & Environment Alliance, Belgium; 2 Faculty of Medicine, University of Novi Sad, Novi Sad, Serbia; 3 Institute of Public Health of Vojvodina, Novi Sad, Serbia; 4 School of Public Health, Université Libre de Bruxelles, Brussels, Belgium; 5 Clinical Center of Vojvodina, Centre for Radiology, Novi Sad, Serbia; 6 Institute of Lowland Forestry & Environment, University of Novi Sad, Novi Sad, Serbia

## Abstract

COVID-19 pandemic yet again showed that health crises and epidemics are introducing urban planning as a public health response. Globally, we saw a renewed interest in urban environment and healthy living and the changes in urban environments which can make for a healthier living. Even before the pandemic, various urban concepts and models that take as basis a health-oriented, holistic approach are being implemented in many cities. To name a few: car-free centres or neighbourhoods, the so-called ‘Superblocks’, neighbourhoods with low-speed traffic, walkable and cyclable cities aiming at all amenities being easy reach so-called ‘15 Minutes city’. COVID-19 crisis only accelerated many of these initiatives and brought them to global level need and attention. Such interventions are being introduced to demotivate the use of polluting cars, to ease up and to promote healthy and active transportation such as walking and cycling. As a consequence, those interventions not only are hoped to lead to an increase in physical activity, but also better air quality, reduction of noise. Cities have accelerated urban transformations of the space for active transportation such as the introduction of more cycling lanes in their networks, transforming ‘car’ streets to mix use streets, etc. Particularly during the pandemic, the streets that were previously dominated by car use, parking lots, parking spaces, and car lanes have shifted their focus to the pedestrians, healthy and active mobility. Though, not so optimistic continuation of the speed of the changes in urban planning are seen at the end of the pandemic. It is still clear that spaces for people, spaces promoting mental health such as green spaces, green islands, green pedestrian streets and healthy mobility, are missing. Lockdown measures of reducing the car traffic and increasing the walkable spaces for citizens were primarily imposed to save public health but had one important co-benefit - improved air quality in many areas.

